# Editorial: Novel Insights Into a Functional HIV Cure

**DOI:** 10.3389/fmicb.2021.797570

**Published:** 2021-12-01

**Authors:** Luca Sardo, Cristina Parolin, Takeshi Yoshida, Alfredo Garzino-Demo, Taisuke Izumi

**Affiliations:** ^1^Department of Infectious Disease and Vaccines, Merck & Co., Inc., Kenilworth, NJ, United States; ^2^Department of Molecular Medicine, University of Padua, Padua, Italy; ^3^Department of Molecular Virology, Tokyo Medical and Dental University (TMDU), Tokyo, Japan; ^4^Department of Microbiology and Immunology, Institute of Human Virology, School of Medicine, University of Maryland School of Medicine, Baltimore, MD, United States; ^5^Department of Biological Sciences, Misher College of Arts and Sciences, University of the Sciences in Philadelphia, Philadelphia, PA, United States

**Keywords:** human immunodeficiency virus (HIV), functional cure, latency, latency reversing agents (LRA), immunotherapy

## Introduction

Although efficient suppression of HIV replication below the limits of detection is achievable with potent combination antiretroviral therapy (cART), the risks of adverse effects and drug resistance still exist. HIV latently infected cells are in a state of non-productive infection, do not respond to cART and are not cleared by the host immune system. As a consequence, latently infected cells establish reservoirs that persist during cART and become a source of productive infection upon therapy interruption. Various strategies for achieving an HIV functional cure have been proposed, including a kick-and-kill approach using latency-reversing agents (LRA) to reactivate the reservoir, a block-and-lock strategy to permanently silence provirus transcription, and others that aim to induce immune control after cART interruption (Zerbato et al., 2019; Li et al., 2021; Ward et al., 2021). We hereby invited interdisciplinary researchers to bring their scientific knowledge and observations to advance novel treatment paradigms toward achieving cure.

## Innate Immunity and HIV Reservoir Cells

Acquired resistance to type I IFNs can occur both in HIV actively and latently infected cells, contributing to the maintenance of the reservoir. Sundaraj et al. demonstrated an intriguing connection between the IFN pathway and HIV protease activity. IFN signaling was inhibited via the HIV protease-mediated cleavage of TANK-binding Kinase 1, which prohibited IFN regulatory factor 3 phosphorylation. Hendricks et al. highlighted how HIV evolution generates an immunologic sanctuary that sustains persistent virus infection in macrophages avoiding IFN-mediated antiviral responses.

## Acquired Immunity

Jones et al. examined a cohort of people living with HIV (PLWH) who have consistently low viral loads. The authors analyzed various immune cell populations isolated from these individuals' PBMCs before and after cART initiation and revealed that CD8+ Cytotoxic T lymphocytes (CTL) were critical to controlling HIV replication. HIV Gag is widely considered a potent antigen to induce robust HIV-specific CTL and is the target for developing an anti-HIV vaccine (Edwards et al., 2002; Zuñiga et al., 2006; Kiepiela et al., 2007). Olusola et al. reported non-synonymous mutations in functionally conserved HIV Gag epitopes in blood isolated from PLWH. Since several escape mutations were found in the epitopes of HIV DNA vaccine design, the authors suggested that slight modifications should be required for further HIV Gag-derived vaccine development. Ikeda et al. reviewed the potential role of the antiviral host protein, APOBEC3, for both innate and adaptive anti-HIV immunities. While APOBEC3G induced premature termination codons, it could also enhance the abundance of HIV-derived epitopes and activation of HIV-specific CTL. The sublethal mutations may contribute to the generation of CTL escape variants due to the changes in the CTL epitopes and flanking regions.

## Host Virus Interactions and Cure Strategies

A long-standing approach for cure in the cART era is to purge latently infected cells that persist during therapy. Matsuda et al. proposed using the DAG-lactone YSE028 as an LRA and evaluated its ability to synergize with other LRAs to selectively activate mechanisms of cell death in infected cells. Janssens et al. reviewed the state of the art on the importance of proviral DNA integration sites and relative chromatin organization for HIV cure. Viral DNA integration sites are shifted toward the inner nucleus from the nuclear periphery in close association to the nuclear pore by inhibiting Integrase-LEDGF/p75 interaction with inhibitors (LEDGINs), with a consequent larger population of the provirus exhibiting transcriptional silence. Pasternak et al. discussed how the chromatin context of the proviral integration sites and the number of intact proviruses that contribute to the replication competence of the reservoir likely influence latent proviruses reactivation capacity. A small HIV reservoir size is necessary but insufficient for post-treatment remission to achieve a functional HIV cure. Therefore, complementary biomarkers would be required to accurately predict post-treatment HIV remission.

## Methods for Examining HIV Reservoirs

Quantifying the size of the long-lived reservoir of cells carrying replication-competent HIV is essential to measuring the impact of curative efforts. Wu et al. developed a novel immunoassay combining immunoprecipitation and a digital ELISA method with a single molecule array to improve the measurement of HIV and SIV Gag proteins from biological samples, particularly at low concentrations. Recent intriguing reports (Imamichi et al., 2020) revealed that a portion of the defective reservoir is translationally competent and can generate structural proteins, potentially producing virus-like particles (VLPs). The immunogenicity of VLPs is affected by their maturation status and can be relevant to sustain chronic immune stimulation. Sarca et al. focused on developing a fluorescence-based microscopy tool to rapidly screen virus maturation with high throughput and unbiased computational analysis.

## Summary

The HIV field has successfully advanced antiretroviral therapies that allow a near-normal life expectancy for PLWH. However, a strategy to achieve viral control in the absence of cART remains an unmet challenge. The main obstacle in achieving a functional cure is ascribed to the integration of the proviral DNA in the host genome and the formation of reservoirs of infected cells that persist even during cART. Although significant progress has been made, questions remain about the major cell types contributing to persistent reservoirs and their anatomical localization, possibly indicating that HIV persistence is established through multiple mechanisms. Persistent immune stimulation in PLWH on cART remains a health concern as inflammatory cytokines are elevated by HIV infection, likely in response to expression of viral antigens in transcriptionally and translationally active proviruses found in the reservoir. In this Research Topic, we focused on HIV persistence and reached the conclusion that a combination of multiple cure strategies, including kick-and-kill, block-and-lock, and immunotherapies may be required to achieve a functional cure. Recognizing that HIV remains a chronic disease, understanding the events that sustain persistence and developing therapies targeting the elimination of infected cells remain keys toward cure and remissions strategies.

## Author Contributions

LS and TI contributed to formulating this Research Topic theme, inviting authors, and collecting articles. All authors contributed to acting as handling editors for the submitted manuscripts and writing the editorial article.

## Funding

This project was supported in part by start-up from the University of the Sciences in Philadelphia to TI.

## Conflict of Interest

LS is employed by Merck & Co., Inc. The remaining authors declare that the research was conducted in the absence of any commercial or financial relationships that could be construed as a potential conflict of interest.

## Publisher's Note

All claims expressed in this article are solely those of the authors and do not necessarily represent those of their affiliated organizations, or those of the publisher, the editors and the reviewers. Any product that may be evaluated in this article, or claim that may be made by its manufacturer, is not guaranteed or endorsed by the publisher.
